# Supplementary material for: Plumbagin-induced oxidative stress leads to inhibition of Na+/K+-ATPase (NKA) in canine cancer cells
Source: Sci Rep. 2019 Aug 7;9:11471. doi: 10.1038/s41598-019-47261-x (PMC6685937; doi:10.1038/s41598-019-47261-x)
Supplement: Supplementary file 1 — Full blots of cropped images from Fig. 2C are shown. [file 41598_2019_47261_MOESM1_ESM.pdf]

Supplementary Figure 1

Manuscript title: Plumbagin-induced oxidative stress leads to inhibition of Na^+/K^+-ATPase (NKA) in canine cancer cells.

Author list: Yousef Alharbi, Arvinder Kapur, Mildred Felder, Lisa Barroilhet, Timothy Stein, Bikash R. Pattnaik, Manish S. Patankar

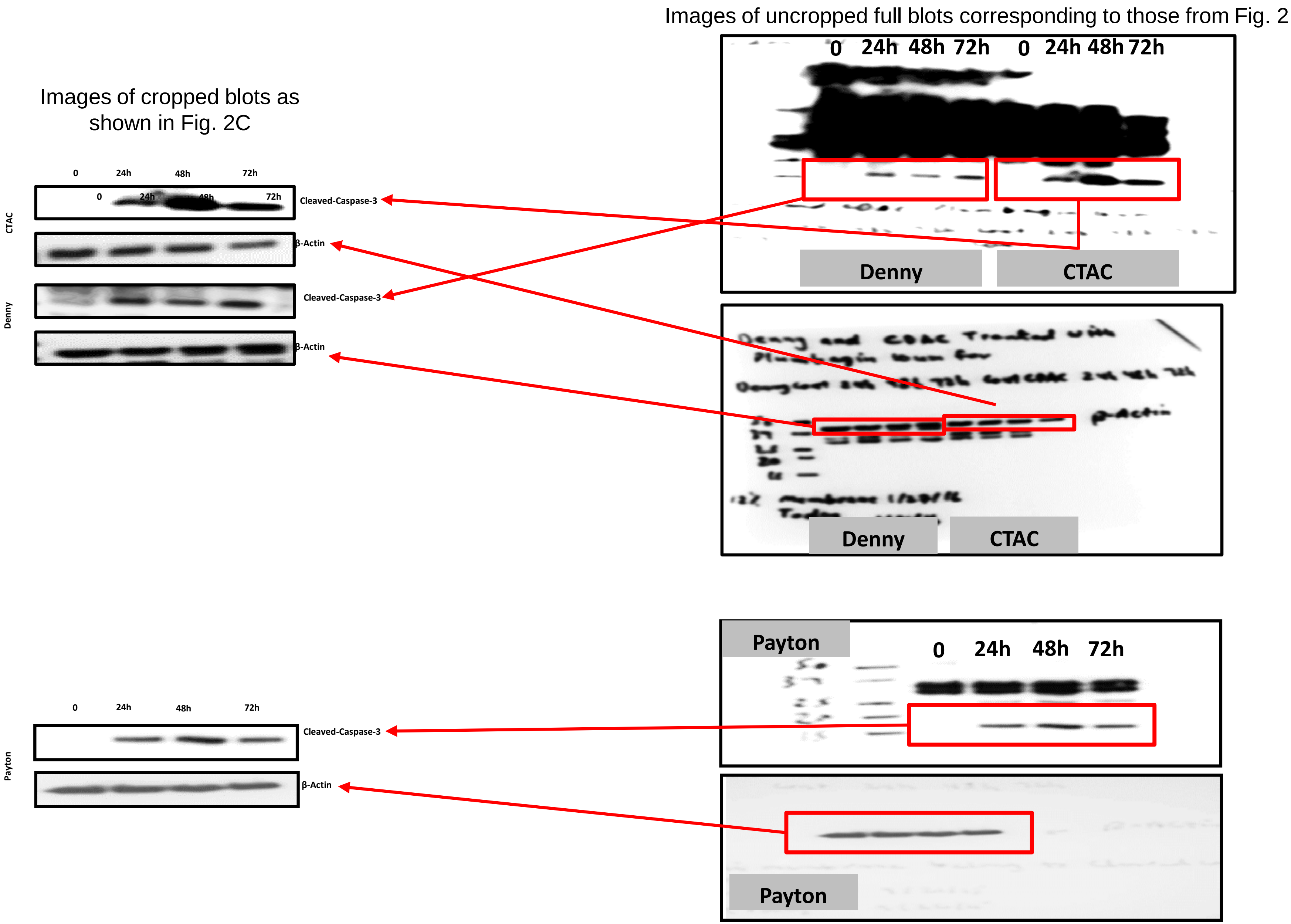

Supplementary Figure 1. Full blots of cropped images from Fig. 2C are shown. For cleaved caspase 3, we used a rabbit anti-human antibody. This antibody is not optimized for detection of canine cleaved caspase 3. Therefore, it was necessary to overexpose the blot and to use the highly sensitive WestFemto (ThermoFisher) kit for detection of the bands. As a result, in CTAC and Denny the pro-caspase3 bands are highly overexpressed so that we could clearly visualize the cleaved caspase 3 bands. The actual Fig 2 is included for reference and red arrows and red boxes indicate the cropped regions shown in Fig. 2C for each cell line and protein analyzed.
